# Understanding Issues Faced by West Midlands’ International Medical Graduates (IMG) Psychiatry Trainees and How to Support them

**DOI:** 10.1192/bjo.2024.288

**Published:** 2024-08-01

**Authors:** Supriya Dastidar, Oluwadamilola Ogunsina, Asma Javed

**Affiliations:** 1 Birmingham and Solihull Mental Health NHS Foundation Trust, Birmingham, United Kingdom; 2 Black Country Healthcare Foundation Trust, Dudley, United Kingdom; 3 Black Country Healthcare NHS Foundation Trust, Dudley, United Kingdom

## Abstract

**Aims:**

The GMC 2023 workforce report indicates that doctors with primary medical qualification (PMQ) outside United Kingdom (UK) made up 62% of new additions to the register in 2022, with international medical graduates (IMGs) from outside the European Economic Area accounting for a further 10%. In 2023, 49.8% of psychiatry trainees in West Midlands were IMGs.

We have enough evidence to show that IMGs experience significant differential attainment in both training and exams. They also have an added burden of adjusting to a new country, language, culture, and society, not to mention adapting to a novel medical system and work culture. Attempts have been made to address this through induction, clinical supervision, etc.

This survey aims to understand the challenges faced by West Midlands psychiatry IMG trainees and to identify how best to support their needs.

**Methods:**

A questionnaire survey was designed using the Microsoft forms platform and disseminated via the West Midlands School of Psychiatry in October 2023 to all trainees whose PMQ was outside UK. The survey gathered feedback on quality of inductions received, clinical supervision, difficulties experienced in training/examinations and awareness of available IMG-specific resources.

**Results:**

36 trainees with PMQ from 14 countries outside the UK completed the survey. 31% of the respondents were CT1 trainees. 17% had less than a year of NHS experience. All respondents had attended their current job induction. 64% rated their workplace induction as ‘Good’ or above, 50% rated trust and deanery induction at ‘Good’ or above. Only 17% of respondents had received IMG-specific induction. Many felt that induction was an information overload in a short space of time. 83% received weekly, hourly supervision. 69.4% rated support from their supervisor as ‘Very good’ or above. Respondents reported difficulties in immigration, finances, systemic racism, cultural and language adaptation. Other difficulties include portfolio, research experience and audits. MRCPsych exam difficulties were reported in 46% respondents especially around study materials and preparation. Trainees wanted IMG specific induction and supervision, pastoral care, portfolio support, MRCPsych exam support, mentoring, guidance around career progression and research.

**Conclusion:**

The survey results show that IMG trainees do not receive appropriate and necessary IMG-specific induction and supervision even though they make up nearly half of the trainee cohort. The Deanery, NHS trusts and clinical supervisors can utilize the results of this survey to inform strategies to support IMGs better. Focus groups are due to be held shortly to get further qualitative feedback.

